# DprA/Smf protein localizes at the DNA uptake machinery in competent *Bacillus subtilis *cells

**DOI:** 10.1186/1471-2180-7-105

**Published:** 2007-11-28

**Authors:** Serkalem Tadesse, Peter L Graumann

**Affiliations:** 1 Institut für Mikrobiologie, Faculty for Biology, Schänzlestr. 1, Albert-Ludwigs Universität Freiburg, 79104 Freiburg, Germany; 2 Current address : Yale University, School of Medicine Department of Therapeutic Radiology New Haven, 06510 CT USA

## Abstract

**Background:**

DprA is a widely conserved bacterial protein and has been shown to confer an important function during transformation in competent cells, possibly through protection of incoming DNA. *B. subtilis *DprA (called Smf) and has been shown to play an important role during transformation with chromosomal DNA, but its mode of action is unknown.

**Results:**

We show that *B. subtilis *DprA/Smf is more important for transformation with plasmid DNA than with chromosomal DNA. A functional Smf-YFP fusion localized as discrete foci to the cell pole in a subset of cells grown to competence, dependent on the ComK master transcription factor. Smf-YFP foci colocalized with ComGA-CFP. However, a considerable number of cells having high ComK activity contained Smf dispersed throughout the cytosol and lacked a polar Smf assembly. The absence of polar Smf-YFP foci in these cells strongly correlated with the absence of ComGA-CFP foci, and *comGA *mutant cells mostly lacked polar Smf-YFP foci. Smf formed polar assemblies in the absence of RecA, and RecA formed dynamic threads after addition of DNA in a *smf *deletion strain. Upon addition of DNA, Smf-YFP foci relocalized from the poles to the cell centre, dependent on the presence of RecA protein.

**Conclusion:**

Our data show that Smf is recruited to the polar competence machinery, and that polar Smf assembly requires a functional DNA uptake complex. High ComK levels drive expression of Smf in 20% of all cells grown to competence, but not all competent cells contain a polar DNA uptake machinery, showing that ComK activity is necessary but not sufficient to achieve assembly of the uptake machinery in all cells. Smf and RecA localize independently of each other, in agreement with our finding that Smf is much more important for plasmid transformation than RecA, but RecA influences the dynamic localization pattern of Smf. Our data show that DprA/Smf acts downstream of the DNA uptake machinery, and support the idea that Smf protects incoming ssDNA, possibly in conjunction with RecA.

## Background

Bacterial competence describes the physiological state that permits the uptake of exogenous DNA in macromolecular form for integration into the chromosome via homologous recombination [1,2]. Competence has been found in a range of bacterial phyla, including the Gram positive *Bacillus subtilis*. Because many pathogenic bacteria have the ability to gain competence, this process is important in light of spreading of antibiotic resistance genes. *B. subtilis *cells develop competence when they enter the stationary phase of growth in response to nutrient limitation [1,2]. During this developmental process, only a fraction of the cells transiently differentiate into competent cells that have physiological characteristics different from those of non-competent cells. This phenomenon is called bistability, in which cells of a single population switch reversibly between two transcriptional states [3-5]. In *B. subtilis*, 10–20% of the cells in a given population differentiate to the competence state, which lasts for 3–5 hours, after which cells turn of transcription of competence genes and exit the so called K-state [2,6].

The master competence transcription factor, ComK, regulates at least 165 genes during competence [6-8], and has been shown to become active exclusively in only those few competent cells (10–20%) [9,10]. At least 16 competence proteins are needed to bind environmental DNA to the cell membrane and transport this DNA into the cytosol, where it is available for recombination with resident DNA [11]. Within the competence machinery, proteins generally work at three different levels. The process of binding DNA at the surface of the cell requires a pseudopilus-like structure (possibly guiding DNA through the peptidoglycan layer) and a DNA-binding protein (ComEA) [12], and is reversible [13]. Transport of DNA is mediated through a channel in the cell membrane (ComEC) [14], and is highly powerful and processive [13]. DNA transport depends on the proton motive force [13], and may be driven by an additional energy source. The transport subdivision includes ATPases, like ComGA [15] or ComFA [16,17], which may enable coupling of ATP hydrolysis and transport. The third group of proteins in the machinery are cytosolic proteins such as RecA, and RecN, that interact with ComGA [18] and mediate homologous recombination, and proteins of unknown function such as YwpH/SsbB, which is homologous to ssDNA-binding SSB (SsbA) protein [17].

Interestingly, the competence machinery assembles at a single cell pole in competent *B. subtilis *cells. This includes the DNA uptake machinery [17] and three cytosolic proteins, RecA, RecN and YwpH [17,18]. Upon addition of DNA to the cells, RecA protein forms dynamic filamentous structures, termed threads, which are thought to guide ssDNA that is taken up through the ComEC channel to the nucleoid. Here, RecA mediates strand exchange between the chromosomal DNA duplex and taken up DNA, given that extensive homology exists between ssDNA and the chromosome [18]. Thus, competence in *B. subtilis *is a temporally and spatially highly organized process.

DprA (DNA processing protein) is a widely conserved protein involved in competence in *Hemophilus infuenzae*, *Helicobacter pylori *and *Campylobacter jejuni*, and is called Smf in *B. subtilis *or CilB or Dal in *Streptococcus pneumoniae*. DprA orthologs have been shown to impart an important role in transformation with chromosomal DNA or with plasmid DNA [6,7,19-21], and the *S. pneumoniae *ortholog is thought to protect incoming DNA during transformation [22], but genetic and biochemical functions of DprA/Smf are not yet understood. Here we investigate *B. subtilis *Smf using genetic, cell biological and biochemical tools. *B. subtilis *DprA/Smf is important for transformation with chromosomal DNA, but even more so with plasmid DNA. DprA/Smf shows localization characteristics similar to but also distinct from representatives of the two groups of proteins of the competence machinery that play a major role in DNA uptake (ComGA) and DNA transformation (RecA and RecN), suggesting that DprA/Smf plays an important role at the interface between the uptake and the recombination machineries.

## Results

### DprA/Smf plays an important role in transformation of chromosomal DNA and of plasmid DNA

To obtain more information on the function of *B. subtilis *Smf (DprA), we tested an *smf *deletion strain for transformation efficiency with different kinds of DNA. As was reported before, the deletion strongly reduces transformation efficiency with chromosomal DNA (10-fold reduction compared with wild type cells, Table 1), but has an even more severe effect on the ability of cells to be transformed by plasmid DNA, be it a bona-fide self replicating plasmid (60-fold reduction), or a *E. coli *plasmid with a region of homology for chromosomal integration (20-fold reduction, Table 1). Thus, like *H. pylori *DprA, *B. subtilis *Smf/DprA is important for transformation with plasmid DNA, even more so compared with transformation by chromosomal DNA. Smf/DprA plays a more important role during transformation with a self replicating plasmid than RecA (transformation efficiency is 30-fold lower in the *smf *mutant compared to the *recA *mutant strain), but is less important for transformation with chromosomal DNA than RecA (efficiency of *recA *mutants is > 700 fold or 70 fold reduced compared with wild type or Δ*smf *cells, respectively) (Table 1). To find out if Smf/DprA affects the same pathway during transformation than RecA protein (i.e. homologous recombination), we created a *smf recA *double mutant strain. Transformation efficiency of a (self replicating) plasmid remained similarly low like in the single Δ*smf *cells, and transformation efficiency of a *E. coli *(integrative) plasmid for the double mutant was less than 2 fold reduced compared to the *recA *or *smf *single mutant strains, respectively, which are already severely deficient in transformation (Table 1). It should be noted that PY79 *recA *mutant cells have a much higher transformation efficiency than other *B. subtilis *strains, possible due to the presence of prophages than can supply recombination activity. Our data show that Smf and RecA are both important for transformation, but in a different manner with regard to plasmid or chromosomal DNA.

**Table 1:** Transformation efficiency of mutant cells compared to wild type cells

Strain	Chromosomal DNA	*E. coli *plasmid (integrative)	Plasmid (self replicating)
Wild type	100	100	100
ST21 (Smf-YFP)	79.5 (± 4.5)	102 (± 5)	96.8 (± 3.5)
Δ*smf *(*smf::Ery*)	10.8 (± 2.0)	0.58 (± 0.25)	1.69 (± 0.25)
Δ*recA *(*recA*::*Cm*)	0.11 (± 0.05)	0.49 (± 0.05)	49.5 (± 2.5)
Δ*recA*, Δ*smf*	0.08 (± 0.04)	0.24 (± 0.06)	0.94 (± 0.45)

Transformation efficiency was determined by counting number of transformants versus number of viable cells (in triplicate). Numbers are percent relative to wild type (PY79) cells.

### Smf does not influence the localization of ComGA, or RecA or RecN dynamics

**Figure 1:**
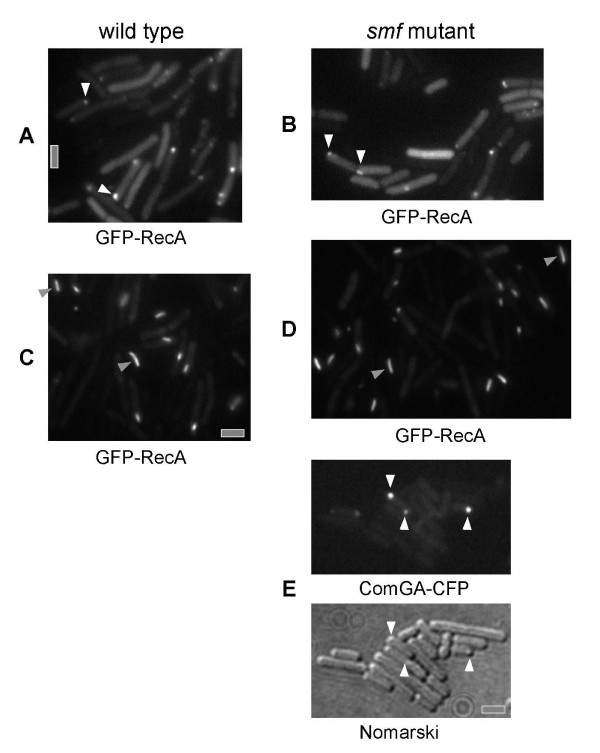
Fluorescence microscopy of *B. subtilis *cells grown to competence. A) Wild type cells expressing GFP-RecA, B) Δ*smf *(*smf*:: pMutin-smf) cells expressing GFP-RecA, C) Wild type cells expressing GFP-RecA 30 min after addition of chromosomal DNA, D) Δ*smf *(*smf*:: pMutin-Smf) cells expressing GFP-RecA 30 min after addition of chromosomal DNA, E) Polar localization of ComGA-CFP in Δ*smf *cells. Lower panel shows acquisition of the outlines of cells in Nomarski DIC. White arrowhead indicate the polar position of ComGA-CFP in Fig 2E and GFP-RecA in Fig 2B & C; Grey arrow heads indicate the thread like pattern of GFP-RecA after addition of DNA; All images were equally scaled; grey bars 2 μm.

Possibly, Smf affects the assembly of the polar DNA uptake machinery. To investigate this idea, ComGA-CFP, which is a component of the polar DNA uptake machinery [17], was visualized in *smf *mutant cells. No difference in the pattern of ComGA-CFP localization could be observed between wild type and *smf *mutant cells (13% of wild type cells or 12.5% of *smf *mutant cells showed polar ComGA-CFP foci) (Figure 1E, 300 cells analysed, and data not shown), suggesting that Smf is not involved in the polar recruitment of one component of the uptake machinery. However, these experiments do not rule out the Smf affects assembly of the complete uptake complex that consists of at least 9 proteins [11].

To find out if Smf affects the function of RecA during transformation in competent cells, we monitored the localization of GFP-RecA in *smf *mutant cells grown to competence. Similarly to wild type cells, GFP-RecA localized to the cell poles in 20% of the cells in the absence of Smf (Figure 1B, compare with 1A), and formed dynamic threads after addition of DNA, irrespective of the presence or absence of Smf (Figure 1D, compare with 1C). Thus, the two important forms of RecA – accumulation at the competence cell pole and formation of threads – operate apparently normally after loss of Smf protein. Similarly, RecN-YFP localized to a single cell pole in *smf *mutant cells (data not shown). These experiments show that Smf does not act directly upstream of RecA or of RecN, and support the idea that Smf and RecA perform somewhat distinct functions with regard to transformation. Because formation of RecA threads depends on DNA uptake, the results suggest that Smf acts after DNA uptake, but not downstream of RecA during homologous recombination.

### Smf localizes throughout the cytosol in competent cells, but also as discrete foci at a single cell pole in most but not all competent cells

**Figure 2:**
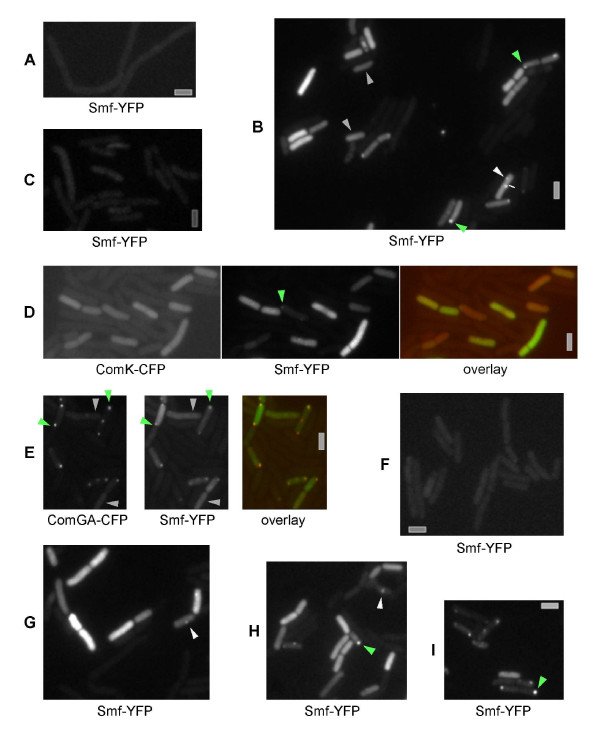
Fluorescence microscope of *Bacillus subtilis *cells carrying Smf-YFP. A) Strain ST21 growing exponentially, B) ST21 grown to competence, C) sporulating ST21 cells, D) strain ST21 grown to competence. First panel shows CFP channel, second panel shows YFP channel, third panel show overlay of ComK-CFP (red) and Smf-YFP (green). E) Strain ST33 grown to competence. First panel CFP, second panel YFP, third panel overlay of ComGA-CFP (red) and Smf-YFP (green). F) Total absence of both polar and cytosolic Smf-YFP localization in Δ*comK *cells treated like wild type cells grown to competence (strain ST32). G) few Δ*comGA *cells grown to competence contain polar Smf-YFP foci (strain ST34), H) few Δ*comEC *cells grown to competence have polar Smf-YFP foci (strain ST35), I) Cells expressing Smf-YFP in a *recA *null background (strain ST 36). Green arrowheads indicate the polar position of Smf-YFP in (B), (D), (H), and (I), and polar Smf-YFP and ComGA-CFP in (E); grey arrowheads indicate cytosolic Smf-YFP without polar foci; white arrowheads indicate central foci of Smf-YFP; grey bars 2 μm.

The expression of Smf has been shown to be > 20 fold induced during competence, dependent on the ComK master regulator [6-8]. To test these findings in live cells, we generated a Smf-YFP fusion that is expressed as sole source of Smf under control of the original promoter, and is fully functional, as judged from its ability to have a transformation efficiency that is indistinguishable from that of wild type cells (Table 1). Only full length Smf-YFP protein was produced, as analysed by Western blotting (data not shown). Smf-YFP fluorescence was only visible in 20% of all cells grown to competence (Figure 2B, 400 cells analysed), but neither in exponentially growing cells (Figure 2A), nor in sporulating cells (Figure 2C). No fluorescence was observed in cells grown to competence the absence of ComK (Figure 2D, 250 cells analysed). Interestingly, two different patterns of localization were apparent: 11% of all cells grown to competence contained a low level of fluorescence throughout the cells and clear fluorescent Smf-YFP foci. Most of the foci were present as single cell pole foci in 10% of the cells, and 1% contained several foci (> 400 cells scored). On the other hand, 9% of all cells had a high level of fluorescence throughout the cytosol, and lacked any visible foci (foci were on average 24% brighter than high-level cytosolic fluorescence, a difference that is easily picked up by the camera, so the foci are not masked by cytosolic Smf-YFP signals). These data suggest that Smf is a soluble cytosolic protein rather than a strictly membrane-associated protein. We used time lapse microscopy to study if the Smf-YFP foci are static, similar to GFP-RecA foci, or if they are dynamic, like RecN-YFP foci [18]. Smf-YFP foci did not show any considerable movement between 1 min intervals in 55 cells monitored, but remained at their polar (or occasionally central) location for at least 15 minutes (data not shown). To test if Smf-YFP fluorescence correlates with the expression of ComK-CFP, we combined both fusions and found that Smf-YFP fluorescence throughout the cells or Smf-YFP foci are only observed in cells having high ComK-CFP fluorescence (Figure 2E). These results verify that Smf is a ComK dependent competence-specific protein, and that Smf has a unique pattern of subcellular localization.

### Smf colocalizes with ComGA at the cell pole, independent of RecA, and localizes throughout competent cells lacking polar ComGA protein

To find out why Smf has two distinct patterns of localization, we combined the Smf-YFP fusion with a ComGA-CFP fusion. ComGA-CFP localized to a single cell pole in competent cells in 13% of all cells grown to competence, as previously reported (Figure 2F). In 97% of these cells (i.e. 12.5% of all cells), polar ComGA-CFP foci colocalized with Smf-YFP foci, while 3% of these cells showed cytosolic localization of Smf-YFP and polar ComGA-CFP localization (Figure 2F, > 350 cells analysed). Intriguingly, an additional 9% of all cells grown to competence contained high Smf-YFP fluorescence throughout the cells, but these did not contain ComGA-CFP foci (Figure 2F). Therefore, Smf-YFP foci strongly correlate with the presence of ComGA-CFP foci, and thus with the presence of the competence machinery, while in the absence of the polar complex, Smf-YFP is dispersed throughout the cell. To obtain further information, we visualized Smf-YFP in competent *comGA *mutant cells. Smf-YFP foci were present in only 0.7% of the mutant cells grown to competence, whereas 17% of the cells had high Smf-YFP levels throughout the cells (with 350 cells analysed; in about ^1^/_3 _of these cells, Smf frequently localized in patches or foci, rather than uniformly throughout the cytosol) (Figure 2G). Thus, the presence of ComGA is important, but not required, for the formation of polar Smf-YFP foci. We also monitored the localization of Smf-YFP in cells lacking the ComEC DNA uptake channel. Only 2% of *comEC *mutant cells contained polar Smf-YFP foci, while 18% of all cells grown to competence contained solely cytosolic Smf (340 cells analysed) (Figure 2H). Therefore, the presence of the competence machinery rather than specifically ComGA or ComEC is required for efficient recruitment of Smf to the cell pole. It is possible that another protein of the competence complex serves as a specific binding partner for Smf. In any event, our results also show that several competent cells (that is cells with sufficient ComK activity to drive expression of Smf) do not contain a fully assembled polar competence machinery, showing that high ComK activity is not sufficient to grant the assembly of the polar DNA uptake machinery.

To further investigate determinants of polar localization of Smf, we moved the Smf-YFP fusion into *recA *or *recN *mutant cells. Smf-YFP foci were still present in cells lacking RecA (Figure 2I) or RecN (data not shown), showing that recruitment of Smf to the pole is independent of the presence of the two homologous recombination proteins. These data support the findings that RecA and Smf localize independently of each other.

### Smf changes its pattern of localization in response to addition of DNA, dependent on RecA

**Figure 3:**
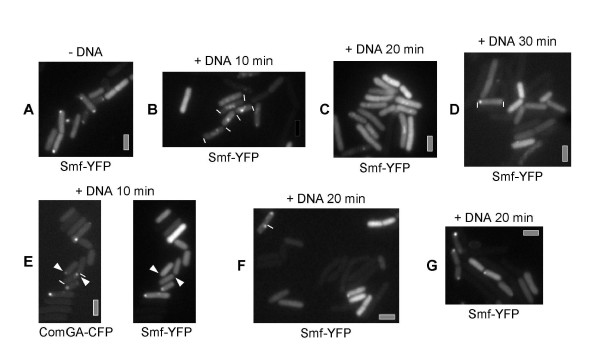
Change in the localization pattern of Smf-YFP. A-D) Strain ST21 (*smf-yfp*) grown to competence, A) without addition of DNA, B) 10 minutes after addition of chromosomal DNA, C) 20 minutes after addition of DNA, D) 30 minutes after addition of DNA; E) strain ST33 (*smf-yfp*, *comGA-cfp*) grown to competence, 20 min after addition of DNA, white triangles indicate cells containing ComGA-CFP foci, but no polar Smf-YFP foci, F) strain ST35/*comEC*::Ery, *smf-yfp*) grown to competence, 20 minutes after addition of DNA. G) strain ST36 (*recA*::*Cm*, *smf-yfp*) grown to competence, 20 minutes after addition of DNA. White lines indicate ends of cells (where these are not easily seen). All images were equally scaled; grey bars 2 μm.

Addition of DNA to competent cells strongly changes the localization pattern of RecA or of RecN. 7% of all cells grown to competence (1/3^rd ^of the competent cells) show GFP-RecA threads after addition of chromosomal DNA, while in the absence of DNA, only 0.5% of the cells contain such filamentous structures [18] (400 cells analysed). These data show that little DNA is present in cells grown to competence, and that addition of DNA induces several fold the formation of RecA threads. We therefore tested if addition of DNA affects the localization of Smf. Addition of plasmid DNA or of chromosomal DNA considerably altered the pattern of localization of Smf-YFP. In the absence of external DNA, Smf-YFP remained as polar foci and/or throughout the cells for at least 3 hours after cessation of growth (Figure 3A). 10 min after addition of self replicating plasmid (0.5 μg/ml) or of chromosomal DNA (0.5 μg/ml), Smf-YFP was still present at the cell pole in only 2.5% of the cells, while 9.5% of the cells showed an irregular staining of mostly one to several foci around the centre of the cells, and 7% showed homogeneous cytosolic localization (Figure 3B, 440 cells were scored for each time point in 3 independent experiments). 20 min after addition of DNA, 1.8% of the cells had polar Smf-YFP foci, while 2.5% of the cells showed an irregular staining of the cell centre (14% had homogeneous cytosolic localization, Figure 3C), until after 30 min, Smf-YFP localized as polar foci in 5% of all cells, while 14% of the cells had a rather homogeneous staining, and 1.8% contained irregular foci (Figure 3D). Although the percentage of polar foci and mid-cell foci varied (by 35%) between individual experiments, addition of DNA reproducibly led to the formation of intracellular foci and decrease in the percentage of polar foci. One has to also bear in mind that changes in Smf-YFP localization patterns must be seen in relation to the fact that only 20% of the cells contained Smf-YFP signals, and only 12–13% polar Smf-YFP foci, so changes between 12.5 and 1.8% are highly significant. A change in the pattern of localization of Smf-YFP is also evident from the finding that 97% of the cells containing polar ComGA-CFP foci (13% of all cells grown to competence) contained polar co-localizing Smf-YFP foci in the absence of DNA, while 20 min after addition of DNA, only 22% of ComGA-CFP foci-containing cells contained polar Smf-YFP foci in this set of experiments (Figure 3E). Thus, in response to addition of DNA, Smf-YFP transiently forms intracellular assemblies within the cells, and polar foci transiently disassemble in many cells. Possibly, Smf binds to incoming ssDNA, which is transported to the nucleoids for homologous recombination. To test this, we monitored Smf-YFP in *comEC *deleted cells, in which about 2% contain polar Smf-YFP foci in the absence of DNA. Addition of DNA did not change this pattern of localization, 20 min after addition of DNA, 2.1% of the cells contained polar Smf-YFP foci (with 280 cells analysed) (Figure 3F), supporting the idea that incoming ssDNA may be involved in the change of localization of Smf-YFP. However, it is formally possible that the 2% of *comEC *mutant cells showing polar foci are those cells in which polar Smf foci can still be observed after addition of DNA in a wild type culture, so the latter conclusion must be viewed in light of this caveat.

Intriguingly, Smf-YFP did not alter its polar localization pattern after addition of chromosomal DNA in the absence of RecA; between 12 and 13% of *recA *mutant cells grown to competence showed polar Smf-YFP foci between 10 and 60 min after addition of DNA (Figure 3G, 260 cells analysed). Thus, although Smf and RecA localize to the cell pole independently of each other, and RecA forms threads independently of Smf, dynamic relocalization of Smf depends on RecA, showing that a connection exists between the two proteins.

## Discussion

Our work provides important novel insight into the function of DprA (also called Smf or CilB), a protein widely conserved in bacteria, some of which possess natural competence. In competent *S. pneumoniae *cells, the half-life of incoming DNA was strongly reduced in the absence of DprA, and also in the absence of RecA, suggesting that DprA may physically protect incoming DNA before loading of RecA occurs [22]. DprA has been shown to play an important role in the transformation with chromosomal DNA in *B. subtilis *[6,7,19-21]. We have found that transformation with plasmid DNA is even more severely reduced in *smf *(*dprA*) mutant cells compared to transformation with chromosomal DNA. Transformation of plasmids requires the full establishment of the plasmid from single stranded DNA fragments that are taken up by the DNA uptake machinery, and does not involve homologous recombination (HR) with the chromosome, while single stranded chromosomal DNA fragments can be directly used for HR without the need to generate dsDNA intermediates. Our genetic experiments show that DprA/Smf is not as important for transformation with chromosomal DNA as RecA, but more important for transformation with a self replicating plasmid than RecA, suggesting that DprA/Smf and RecA have somewhat distinct functions during transformation. However, these data do not rule out that both proteins act in conjunction. How might DprA confer its important function during competence? Several lines of evidence suggest that DprA/Smf acts at a step directly following DNA uptake. To test if DprA/Smf functions downstream or upstream of RecA, or in conjunction with RecA, we monitored the localization of RecA in the absence of Smf. In competent *B. subtilis *cells, RecA localizes to a single cell pole that contains the DNA uptake machinery [18]. Upon addition of DNA, RecA forms dynamic filamentous structures termed threads that extend from the DNA uptake machinery towards and onto the nucleoids in the cell centre. A RecA allele that is unable to form threads after addition of DNA is non functional, indicating that these structures are active intermediates during transformation. The absence of DprA/Smf did not alter the pattern of localization of RecA, the protein localized to a single pole in competent cells and formed threads indistinguishable from those in wild type cells after addition of DNA. Similarly, Smf did not affect the specific localization of RecN, another proteins involved in HR. Thus, Smf does not appear to act upstream of RecA, but possibly in parallel with RecA. This idea is supported by cytological data gained from the investigation of a fully functional Smf-YFP fusion. Smf-YFP was present throughout the cytosol in about 20% of cells grown to competence. In agreement with DNA array data [6-8], the expression of DprA/Smf was strictly dependent on ComK. However, about 10–12% of the cells containing cytosolic Smf-YFP contained Smf-YFP foci at a single cell pole, which colocalized with ComGA, and thus with the DNA uptake machinery [17]. Thus, DprA/Smf displays two different patterns of localization in competent cells, absence or presence of polar assemblies in parallel with diffusely localized protein. DprA/Smf assembly at the cell pole was strongly reduced in the absence of ComGA or of ComEC. Conversely, Smf-YFP still formed polar foci in cells lacking RecA, showing that Smf and RecA localize independently, but in association with the DNA uptake machinery. Intriguingly, addition of DNA to competent cells strongly influenced the localization of Smf-YFP. Already 10 min after addition of DNA, the number of polar Smf-YFP foci decreased, frequently (but not always) concomitant with the appearance of one or several cytosolic Smf-YFP foci. Between 20 and 30 min after addition of DNA, these assemblies largely disappeared, and Smf became dispersed throughout the cytosol. These data show that Smf is not statically associated with the polar uptake machinery, but can transiently dissociate from the pole. Indeed, we found that purified Smf is a soluble protein, and forms a monomer in solution (data not shown). Interestingly, dynamic relocalization of Smf was dependent on RecA, because Smf remained at the cell pole after addition of DNA in *recA *mutant cells. These data are compatible with and support the idea that DprA/Smf protects incoming ssDNA from nucleolytic attack [22], which will be transported onto the nucleoids for homologous recombination. This function will be important during transformation with chromosomal DNA that can be directly used for integration into the chromosome. Stabilization of incoming ssDNA is probably even more important for establishment of a dsDNA plasmid (requiring ssDNA strand annealing and DNA synthesis), which would explain why DprA/Smf is even more important during plasmid transformation. Our finding that polar Smf foci dissipate upon addition of DNA, dependent on RecA protein, is consistent with our hypothesis that RecA threads are involved in the transport of ssDNA from the pole to the chromosome, and indicates that Smf binds to incoming ssDNA (directly or indirectly) and is transported away from the pole. However, this last idea is still highly speculative. It will be important to find out if DprA/Smf interacts directly with RecA, and to identify other factors that interact with DprA/Smf, to further elucidate its function(s) during transformation.

Our results also provide insight into the assembly of the polar competence machinery. The polar uptake machinery has been shown to transiently assemble and later disassemble at the pole in competent cells, dependent of ComK activity. In our experiments, many cells contained high ComK activity, as well as a high amount of Smf, and thus also of all other competence proteins. However, a considerable proportion of these cells did not contain an assembly of Smf at a cell pole, even after an extended time after the onset of competence, but rather solely Smf located throughout the cytosol. These cells did not contain a fully assembled DNA uptake machinery, as judged from the absence of ComGA in cells lacking a polar Smf assembly. Thus, Smf molecules of the dispersed cytosolic pool relocalize to the cell pole only in cells containing the uptake machinery. These results suggest that high ComK activity is necessary but not sufficient to ensure assembly of the DNA uptake machinery, indicating another level of regulation exists for the assembly of the complete competence machinery. Thus, competence in *B. subtilis *serves as a model for the investigation of assembly of a large membrane-protein complex, and may yield important new insight into this fundamentally important process.

## Methods

### Bacterial strains, growth conditions and transformation

*Escherichia coli *XL-1Blue (Stratagene) was used for cloning experiments grown in Luria-Bertani (LB) rich media supplemented with 50 μg/ml ampicillin. Cells were grown to competence as described previously by Dubnau and Davidoff-Abelson [23], with appropriate antibiotics for selection. Antibiotics were used at the following concentrations: chloramphenicol (Cm) at 5 μg/ml, kanamycin (Kan) at 10 μg/ml, tetracycline (Tet) 10 μg/ml and Erythromycin (Ery) 1 μg/ml.

### Construction of vectors and strains

To create a C-terminal fusion of Smf/DprA with YFP, the 500 bp 3' fragment of the *smf *gene was amplified by PCR using primers 655 and 656 as forward and reverse primers (Table 2), respectively and cloned at *Apa*I and *Eco*RI restriction sites on pSG1164 vector [24]. The resulting plasmid was transformed into wild type *B. subtilis *(PY79), where it integrated at the original locus on the chromosome by single cross-over, resulting in strain ST29. This way, the expression of *smf-yfp *was under the control of the original promoter, and downstream genes could be driven by the xylose promoter. The Smf-YFP fusion is fully functional because the transformation efficiency was comparable to the wild type PY79 strain. Strain ST29 was grown without xylose, because addition of xylose led to aberrant growth of cells, probably because the *topA *gene and the downstream genes were overproduced. ST29 was freshly made competent at all times to see Smf-YFP localization (rather than investigating thawed cells). To create a strain in which ComK activity can be visualized in cells expressing Smf-YFP, strain ST29 was transformed with chromosomal DNA from a strain carrying a fusion of the first five codons of *comK *to *cfp *[10], generating strain ST31. ST29 was also transformed with chromosomal DNA from a strain carrying *comGA-cfp *[17] to give strain ST33. Likewise, Smf-YFP was moved into Δ*comGA *[25] or Δ*comEC *[14] backgrounds to give strains ST34 and ST35. To examine whether Smf has a role in homologous recombination (HR), the Smf-YFP fusion was moved into Δ*recA *or *ΔrecN *backgrounds, to give ST36 and ST38, respectively; GFP-RecA integrated at the amylase locus was moved into the Δ*smf *background (strain 168 *smf*::pMutin-Smf) [7], selecting for spec resistance, to create strain ST37. Strains used in this study are shown in Table 3.

**Table 2:** Primers used in this study

	Names	Sequences 5'-3'
655	*smf *c-ter up	TACGGGCCCCCCCCTGCCGTACTGT
656	*smf *c-ter dwn	CCAGAATTCAAAGGGTTCCGTATATTGAAC
657	*smf*-trunc up	TACGGTACCCCCCCTGCCGTACTGT
658	*smf*-trunc dwn	CCACTCGAGGCCCTGAACGACAATAACG
667 668	Forward *smf *topo Reverse *smf *topo	CACCATGTTGGATCAGGCCGCT TTAATGGTGATGGTGATGGTGAAAGGGTTCCGTATATTGAAC

**Table 3:** Bacterial Strains

Strain	Genotype	Reference
PY79	Wild type	
168 *smf*::pMutin-Smf	*smf*::*Ery*, Em^r^	[7]
ST29	*smf-yfp *(at original locus; Cm^r^)	This work
ST30	*smf-cfp *(at original locus; Cm^r^)	This work
ST31	*smf-yfp*, *comK*-*cfp *(Cm^r^, Kan^r^)	This work
ST32	*comK*::*Ery*, *smf-yfp *(Cm^r^)	This work
ST33	*smf-yfp*, *comGA-cfp *(Cm^r^, Kan^r^)	This work
ST34	*comGA*::Ery, *smf-yfp *(Cm^r^)	This work
ST35	*comEC*::Ery, *smf-yfp *(Cm^r^)	This work
ST36	*recA*::*Cm*, *smf-yfp *(Tet^r^)	This work
ST37	*smf*::*Ery*, *gfp-recA *(pXyl)	This work
ST38	*recN*::*Cm*, *smf-yfp *(Tet^r^)	This work
BG190	*recA::Cm*	[26]
DK37	*gfp-recA::amy *(Spec^r^)	[27]

### Transformation efficiency test

To carry out the plating experiment for transformation efficiency of the different strains, an OD_260 _measurement was taken to determine the concentration of the chromosomal DNA, self replicating shuttle plasmid (pDG145, kanamycin resistance), and integrating plasmid (pYkoV-YFP, chloramphenicol resistance). The measurement of optical density for competent cells was carried out at an OD_600_. After addition of DNA for 30 min, cells were serially diluted and plated onto LB agar having the appropriate antibiotics to be incubated at 37°C for 24 hours. Transformation efficiency was determined as ratio of transformants versus viable cells, and numbers for wild type cells from 3 independent experiments were considered as 100%.

### Image acquisition

Fluorescence microscopy was performed on an Olympus AX70 microscope. The respective competent cells were mounted on agarose gel coated slides. Images were acquired with a digital charge-coupled device camera (Princeton Instruments MicroMax) driven by Metamorph 5.0 program (Universal Imaging Corp., USA). DNA was stained with 4',6-diamidino-2-phenylindole (DAPI; final concentration, 0.2 ng/ml), and membranes were stained with FM-4-64 (final concentration, 1 nM). Filters used were: DAPI – ex360–370, dc400, em420–460, CFP: ex D436/20, dc 455DCLP, em D480/40, YFP: ex HQ500/20, dc Q515LP, em HQ535/30, GFP – ex460–495, dc505, em510–550, FM4–64 ex480–550, dc570, em590. For studies employing addition of DNA to competent cells, 5 μg of chromosomal DNA or 5 μg of plasmid DNA was added to 100 μl of culture containing about 2 × 10^9 ^cells, corresponding to amounts of DNA that give a reasonable number of transformants. Chromosomal or plasmid DNA containing various different resistant genes were employed.

## Authors' contributions

ST performed all experiments and participated in the design of the study and in the writing of the manuscript. PLG conceived of the study, participated in its design and coordination, and wrote the manuscript. All authors read and approved the final manuscript.
